# Mechanisms of artemisinin resistance in *Plasmodium falciparum* malaria

**DOI:** 10.1016/j.coph.2018.06.003

**Published:** 2018-08-01

**Authors:** Niraja Suresh, Kasturi Haldar

**Affiliations:** 1 Boler-Parseghian Center for Rare and Neglected Diseases, University of Notre Dame, 103 Galvin Life Sciences, Notre Dame, IN 46556, USA; 2 Department of Biological Sciences, University of Notre Dame, 103 Galvin Life Sciences, Notre Dame, IN 46556, USA

## Abstract

Artemisinin-based combination therapies (ACTs) have substantially reduced worldwide malaria burden and deaths. But malaria parasites have become resistant to artemisinins. Prior studies suggested two different molecular pathways of artemisinin-resistance. Here we unify recent findings into a single model, where elevation of a lipid, phosphatidylinositol-3- phosphate (PI3P) results in vesicle expansion that increases the engagement with the unfolded protein response (UPR). Vesicle expansion (rather than increasing individual genetic determinants of the UPR) efficiently induces artemisinin resistance likely by promoting ‘proteostasis’ (protein translation coupled to proper protein folding and vesicular remodeling) to mitigate artemisinin-induced proteopathy (death from global abnormal protein-toxicity). Vesicular amplification engages the host red cell, suggesting that artemisinin resistant malaria may also persist by taking advantage of host niches and escaping the immune response.

## Introduction

World-wide levels of malaria have substantially decreased over the last decade. Notably, twenty-one countries are expected to have eliminated the disease by 2020 [1]. Yet a substantial burden remains. In 2016, 445 000 deaths and 216 million new cases were caused by *Plasmodium falciparum*, the most virulent of human malaria parasites [1]. Artemisinin-based combination therapies (ACTs) are frontline, fast acting drugs that have been key to reducing the malaria burden and deaths. However, *P. falciparum* resistance to artemisinins and ACTs has emerged, threatening global malaria control and elimination.

**Figure 1:**
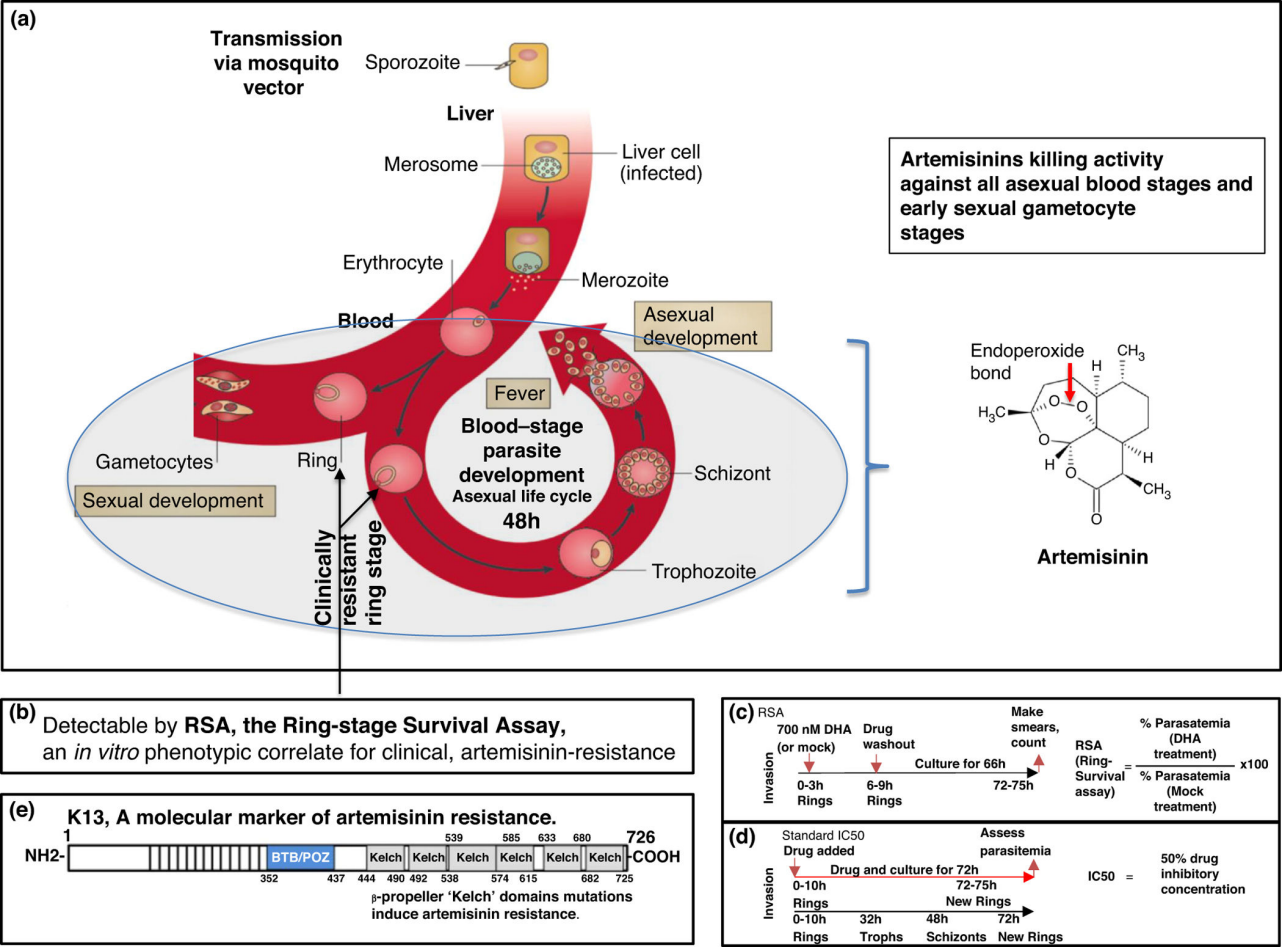
Life cycle of *Plasmodium falciparum* and measures of artemisinin drug resistance. (a) (Adapted from Delves, M., Scheurer, C, *et al*. 2012 [48])*. P. falciparum* malaria infection in humans is initiated when an infected mosquito bites and releases sporozoites that infect liver cells and develop into merozoites. These merozoites emerge into the blood and invade erythrocytes (red blood cells), where they develop over a 48-hour asexual life cycle through morphologically defined ring, trophozoite and schizont stages. Schizont lysis leads to the release of daughter merozoites (which coincides with fever), to initiate a new asexual life cycle. A small subpopulation of ring-form parasites develops into sexual gametocyte stages which are taken up by the mosquito during a blood meal. The killing activity of artemisinins (against asexual and early sexual gametocyte stages) is dependent on the cleavage of their endoperoxide bond. Clinical resistance to artemisinins is seen at the parasite ring stage. (b) Clinical artemisinin resistance is measured *in vitro* by the Ring-stage Survival Assay (RSA). (c) In the RSA, ring-stage parasites 0–3 hour, are treated with maximal concentrations (700 nM) of dihydroartemisinin (DHA, the active form of all artemisinins) seen in plasma for 6 hours, after which the drug is washed out, mimicking pharmacological exposure seen in patients. Parasites are subsequently allowed to progress for another 66 hours, after which parasitemia is determined. The RSA value is calculated as shown. (d) The standard IC50 assay is carried out by exposing ring, trophozoite and schizont stages to continuous drug treatment over 72 hours. (e) *Plasmodium falciparum* K13 (*Pf*Kelch13) is a causal molecular marker of artemisinin resistance. It contains a single BTB domain (common to all Broad-complex, Tramtrack and Bric-a-bric domain (BTB) family proteins), which is present at the amino terminus, followed by multiple copies of β-propeller ‘Kelch’ repeats. K13 contains six β-propeller Kelch domains, mutations in which induce artemisinin resistance.

*P. falciparum* infection in humans is initiated when an infected mosquito bites to release sporozoites that infect liver cells. In the liver, sporozoites develop into merozoites which emerge into the blood stream to invade erythrocytes (red blood cells) and develop through distinct ring, trophozoite and schizont stages over a 48 hour asexual life cycle (Figure 1a). The blood stages of infection cause all of the symptoms and pathologies of malaria (and are thus targeted by all antimalarials in current use). Parasite killing by artemisinins depends on cleavage of their endoperoxide bond (Figure 1a). Proteomic studies indicate that artemisinins alkylate hundreds of proteins [2^●●^,3^●●^] suggesting they may kill by inducing ‘proteopathy’ or global degeneration of the parasite’s (proteinaceous) cytoplasm. Resistance to artemisinins observed in the clinic, is seen in ring parasites that show ‘delayed clearance’ from the circulation, in response to direct administration of drug to patients [4,5]. Trophozoite and schizont stages that sequester in tissues are not found in circulation and hence not detected in blood smears used to monitor parasites [6].

The parasite gene *pfkelch13* (K13) is the primary marker of artemisinin resistance as defined both clinically and *in vitro* using the Ring-stage Survival Assay (RSA; [6] Figure 1b). In the RSA, parasites are treated at maximal concentrations of artemisinins achieved in plasma (~700 nM) for 6 hours to mimic pharmacological drug exposure seen in patients (Figure 1c). Notably, only the earliest stages of rings (0–3 hour in the life cycle) manifest artemisinin resistance, while later stages (including 9– 12 hour and 18–21 hour rings) do not [6]. Moreover, *in vivo* clinical artemisinin resistance cannot be gauged in a standard *P. falciparum* IC50 assay (Figure 1d, carried out over 72 hours of continuous drug pressure for effects on parasite proliferation through the trophozoite/schizont stages).

Based on its sequence and structure, K13 is predicted to a substrate adapter of an E3 ligase [7] (Figure 1e). Its mammalian orthologues (the best characterized of which is Keap1 [7]) target binding, ubiquitination and proteosomal degradation of select substrates, keeping their levels low to maintain proper cellular homeostasis. Mutations in the b-propeller ‘Kelch’ domain diminish targeting and raise substrate levels in a cell. In cancer, K13 orthologues confer resistance to drugs that kill by inducing ‘proteopathy’ in tumors [8]. In *P. falciparum*, two major K13 effector mechanisms have been proposed to overcome artemisinin-induced proteopathy and death. They are namely, first, proteostatic dysregulation of parasite phosphatidylinositol-3- kinase resulting in elevation of parasite PI3P [9] and second, upregulation of parasite oxidative stress and protein damage pathways via the unfolded protein response (UPR) [10]. In this review we discuss the most recent advances and convergence of mechanisms in a unified model of K13-dependent and independent states of artemisinin resistance, with implications for resistance to partner drugs as well as new emergent antimalarials. We conclude by deliberating on how understanding mechanisms of resistance sheds critical insights into challenges that need to be overcome to eliminate parasites from regions with high and low endemicity and rapidly changing rates of transmission of malaria in the world.

## Pathways of artemisinin resistance

The identification of K13 mutations of artemisinin resistance in *P. falciparum* malaria galvanized the identification of underlying mechanisms. Comparative, global transcriptomic profiling of over a thousand clinical strains led to the identification of molecular signatures for the resistant K13 mutant parasites in this set, and implicated upregulation of parasite oxidative stress and protein damage pathways via the UPR, as a mechanism of resistance [10]. Concurrent independent studies using both clinical and laboratory isolates suggested K13 acted as an adapter of PfPI3K in an E3 ligase complex to control protein levels of PfPI3K, in complete absence of changes in *pfpi3k* gene transcript levels [9]. Wild type K13 protein bound PfPI3K catalyzed kinase ubiquitinylation and degradation by the proteasome, while K13 mutants failed to bind, increasing levels of PfPI3K and elevating its lipid product PI3P. Moreover, elevation of PI3P alone efficiently conferred artemisinin resistance [9]. Yet the function of PI3P elevation and its connection to the UPR remained elusive.

**Figure 2:**
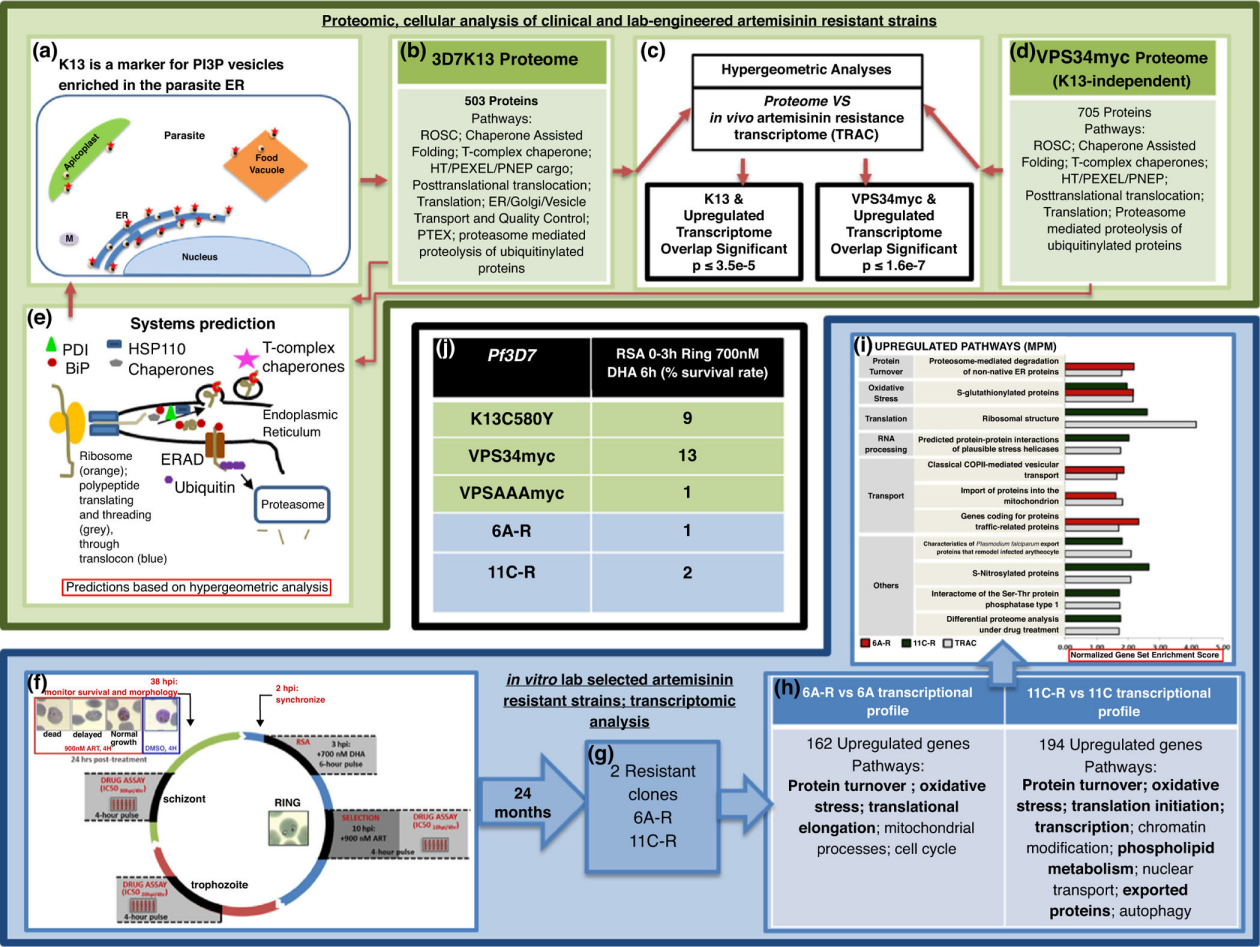
Mechanisms of artemisinin resistance. (a–e) Proteomic and cellular studies of clinical and lab-engineered artemisinin resistance. (*Summarized from* Bhattacharjee *et al*. 2018 [11^●●^]). (a) K13 (red star) is a marker for PI3P (black dots) in vesicles (yellow) enriched in the parasite ER (dark blue). K13-PI3P vesicles are also observed in the food vacuole (orange) and apicoplast (green). These vesicles were not enriched at the parasite mitochondria (M), nucleus or plasma membrane. (b) Biochemical isolation followed by proteomic analysis of the 3D7K13 vesicle revealed several parasite pathways of proteostasis systems enriched in reactive oxidative stress complex (ROSC), Chaperone Assisted Folding; T-complex chaperone; HT/PEXEL/PNEP (host-targeting/plasmodium export element/PEXEL-negative exported proteins) cargo; posttranslational translocation; translation; ER/Golgi/vesicle transport and quality control; plasmodium translocon of exported proteins (PTEX); proteasome mediated proteolysis of ubiquitinylated proteins. (c) Hypergeometric analyses revealed significant overlap between the K13-vesicle proteome and upregulated (but not downregulated) genes of the *in vivo* artemisinin resistant transcriptome (TRAC) [10]. (d) Synthetic resistance by elevation of PI3P induced independent of K13 mutation (using VPS34myc) yielded vesicular proteomes enriched in pathways of ROSC, Chaperone Assisted Folding, T- complex chaperones, HT/PEXEL/PNEP, Posttranslational translocation, Translation, Proteasome mediated proteolysis of ubiquitinylated proteins. Hypergeometric analyses revealed significant overlap between the synthetic PI3P (VPS34myc) proteome and upregulated (but not downregulated) genes of the *in vivo* artemisinin resistant transcriptome (TRAC; c). (e) Vesicular K13 and synthetic PI3P proteomes predict a system of vesicular proteostasis containing oxidative stress responses to protein damage, the UPR and ERAD (ER-associated degradation) pathway including (but not limited to) key ER proteins such as PDI (protein disulfide isomerase), BiP (heat shock protein 70). This model is validated by localization of K13 to vesicles (panel a). (f–i). Transcriptomic studies of *in vitro* lab selected artemisinin resistance (summarized from Rocamora *et al.*, 2018 [12^●●^]). (f) *In vitro* selection of *P. falciparum* clones after long term exposure to artemisinins. The 3D7 strain was exposed at mid ring (10 hour+) to short (4 hour) pulses of a clinically relevant dose (900 nM) of artemisinins continuously for two years. (g) Two *in vitro*-selected resistant clones 6A-R and 11C-R were generated. (h) Their transcriptional profiling showed enhancement of adaptive responses against oxidative stress and protein damage (shown in bold). (i) Normalized Gene Set Enrichment Analysis (GSEA) Score was used to depict overlap between significantly upregulated functionalities of the *in vitro* lab selected resistant lines and *in vivo* artemisinin resistant transcriptome [10]. (j) Ring-stage survival assay (RSA) values associated of indicated strains reported by Bhattacharjee *et al.*, 2018 [11^●●^], Mbengue *et al.*, 2015 [9], Rocamora *et al.*, 2018 [12^●●^].

Two recent studies have directly shed additional light on these pathways, their relatedness and function in resistance [11^●●^,12^●●^]. At the cellular level, high resolution immunoelectron microscopy established that K13 localizes to PI3P vesicles, serving as a marker for these vesicles in the parasite (shown in a schematic in Figure 2a; [11^●●^]). K13-PI3P vesicles reside predominantly in the parasite ER (and at lower levels in the food vacuole and the apicoplast). The major artemisinin resistance mutation K13C580Y quantitatively increased PI3P- vesicles. Biochemical isolation of these vesicles and their proteomic analyses enabled determining their constituent properties and as well as comparisons with the *in vivo* resistance clinical transcriptome. As summarized in Figure 2b, the K13/PI3P vesicle proteome was enriched in multiple proteostasis systems of protein export, quality-control and folding in the parasite ER and cytoplasm including oxidative protein damage, stress and UPR. Hypergeometric analyses (a discrete probability distribution to predict success of outcomes) suggested significant overlap of the vesicle proteome with upregulated (but not downregulated) genes of the *in vivo* artemisinin resistance transcriptome (Figure 2c) reinforcing the association between vesicle amplification and resistance. Synthetic elevation of PI3P (by transgenic expression of human PI3K called VPS34) in absence of K13 mutation, conferred greater than a log increase in artemisinin resistance as measured by RSA and amplified vesicle proteomic signatures of ER proteostasis systems (Figure 2d and e). The proteomes significantly overlapped with adaptive responses to oxidative stress and UPR associated with *in vivo*, clinical artemisinin resistance (Figure 2c). Together, these data suggested that expansion of homeostatic ER vesiculation is a major determinant of artemisinin resistance and likely to be a primary trigger for amplification of pathways of parasite oxidative stress and protein damage pathways via the UPR.

In the second study [12^●●^], *in vitro*-selected artemisinin resistant parasite lines were derived from a laboratory strain (3D7) of *P*. *falciparum* exposed to multiple short pulses of artemisinins during the asexual cycle and grown long term (for 24 months). Two isolated clones without mutation in K13 (Figure 2f–g, clones 6A-R and 11C-R) showed changes of 1.7–2.5 fold increase in artemisinin resistance in a standard IC50 assay (measured over 72 hours) but with low RSA values of 1% (the cut off for resistance) and 2%. Their gene expression profiles showed some enhancement of adaptive responses against oxidative stress and protein damage (Figure 2h) but failed to show significant intersection to the *in vivo* transcriptome (further supporting K13 is associated with clinical artemisinin resistance). A normalized gene set enrichment score (GSEA) was used to capture links in the gene sets of the *in vitro* selected transcriptome to clinical artemisinin resistance at the transcriptional level (Figure 2i). In a modified *in vitro* assay where 10 hour parasites were pulsed for 4 hours (thought to mimic *in vivo* cycles of drug pressure), the *in vitro*-selected strains showed higher tolerance for artemisinin than parental counterparts. *Trans* expression of two top hit genes *PfTrx1* and *PfSpp2* (respectively involved in cellular protection and protein translation) in the parent 3D7 strain, showed a small increase in resistance when measured in 10 hour rings pulsed for 4 hours but did not contribute resistance measurable by RSA (Figure 2j).

## Mechanism of artemisinin resistance

Proteostasis mechanisms of artemisinin resistance are more complex than amplification/mutation of a transporter or enzyme seen in the case of resistance to other antimalarials [13–15]. Consequently, multiple approaches and measurements have been used to assess artemisinin resistance in laboratory studies. Nonetheless, since the RSA provides a gold standard of *in* vitro artemisinin resistance that has been validated against in vivo resistance, it provides a robust index to simplify comparisons between findings across multiple studies. Since variations may arise due to differences in strain background, we used 3D7 strain data to compare the effect of K13C580Y mutation (responsible 80% of resistant parasites in SE Asia) as well as proposed determinants of K13-independent resistance. As shown in panel 2j, transgenic elevation of parasite PI3P (via expression of VPS34) in absence of K13 mutation, induced greater than a log change of resistance in the RSA and comparable to levels induced by K13C580Y. Moreover, expression of VPS34myc amplified a proteome that significantly overlapped with upregulated genes of the in vivo clinical transcriptome (Figure 2d). *In vitro* chemo-selected resistant parasites also independent of K13 showed much lower levels of resistance (RSAs 1–2) and some association with the in vivo clinical artemisinin resistance transcriptome. But transgenic expression of the high value candidate genes of the oxidative stress and protein damage, did not result in an RSA survival rate of 2% (Figure 2j; although they may show a small contribution in a less stringent resistance assay).

**Figure 3:**
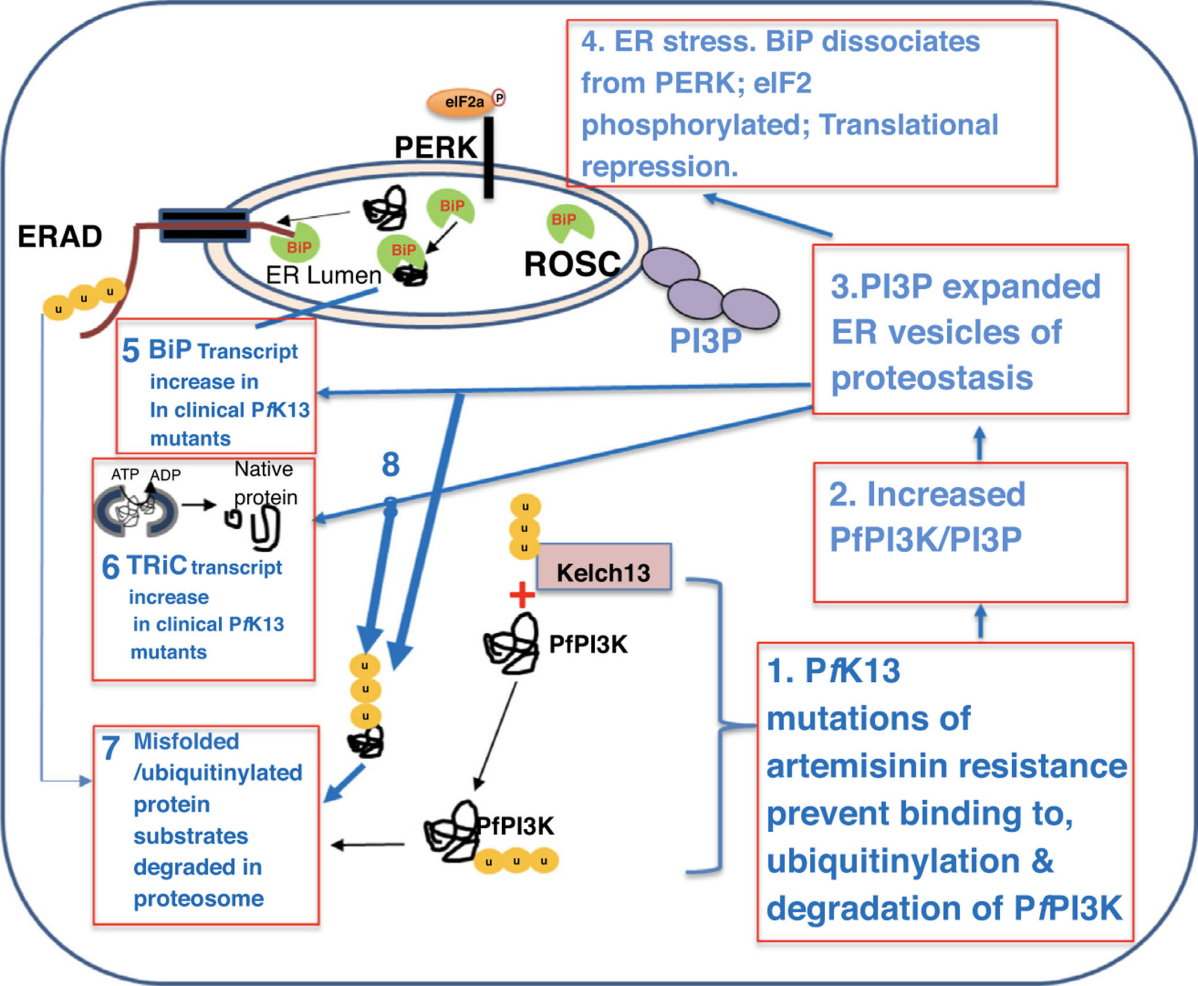
Model unifying proteostasis in ER and cytoplasm of *Plasmodium falciparum* in mechanisms of artemisinin resistance. Pathways of protein quality control in the ER and the cytoplasm stimulate concerted mechanisms of protein translation, translocation, vesicular export and additional chaperone functions to enable proteostasis and thereby restore proper folding of proteins and their function in a cell. A model is proposed for proteostasis pathways to rescue *Plasmodium falciparum* parasites from artemisinin-induced protein damage, proteopathy and death. In artemisinin-resistant parasites, K13 mutations prevent binding, ubiquitinylation and degradation of *Pf*PI3K (step 1, [9]). This leads to an increase in the kinase and thereby its lipid product, PI3P (step 2) causing expansion of homeostatic PI3P-vesicles of proteostasis from the ER (step 3, [11^●●^]) that may underlie a mechanism of autophagy. In addition, these vesicles contain BiP, a key component of the ROSC. BiP is usually bound to UPR transmembrane receptors, but under conditions of stress disassociates from the membrane and binds to misfolded proteins, shifting equilibrium away from and activating UPR receptors. In *P. falciparum*, the UPR receptor, ER transmembrane sensor protein-kinase R (PKR)-like ER kinase (PERK, also known as PK4) has been shown to phosphorylate elongation initiation factor 2a (eIF2a), leading to translational repression, and a reduction of general protein synthesis in artemisinin resistant parasites (step 4, [16^●●^]). Increase in BiP transcript levels *in vivo* artemisinin resistance (step 5) [10], may reflect a response linked to steps 4 and 3. The T-complex protein 1 (TCP1) ring complex (TRiC) chaperone transcript also increased in *in vivo* artemisinin resistance [10] may enable misfolded proteins in the cytoplasm to become properly folded (step 6) appears associated with step 3 [11^●●^]. In the ER-associated degradation (ERAD) pathway misfolded proteins bound and unfolded by BiP (step 5), are translocated to the cytoplasm, ubiquitinylated and degraded in the proteasome (through the ubiquitin-26S proteasome pathway (step 7). Misfolded proteins in the cytoplasm that are not rescued by TRiC maybe ubiquitinylated and targeted for degradation in the proteasome (step 8, [32]).

In sum, amplification of PI3P vesiculation as modeled in Figure 3 (steps 1–3) remains the stronger predictor of artemisinin resistance. Its transcriptional imprint maybe upregulation of ER vesiculation, parasite oxidative stress and protein damage pathways via the UPR. Individual components of adaptive responses against oxidative stress and protein damage may act in concert within PI3P proteostatic vesicles or in addition to them, to confer resistance (Figure 3, steps 4–6). Therefore, although *in vitro***-**selected artemisinin-resistant parasites were not analyzed for levels of PfPI3K-protein or PI3P-lipid vesicles [12^●●^], their transcriptional profiles support the model in Figure 3, that expansion of homeostatic ER vesiculation is a major determinant of artemisinin resistance. The model in Figure 3 also explains additional findings from recent studies that PERK (also known as PK4; step 4) in the presence of artemisinins, phosphorylates eukaryotic initiation factor-2a leading to translation arrest and the induction of latency [16^●●^], providing a high level of proof that that *Plasmodium* manifests at least one (of 3) well- defined effector arms of the UPR of eukaryotes in artemisinin resistance.

In higher eukaryotes, PI3P expansion in the ER stimulates autophagy to efficiently remove misfolded/toxic protein aggregates [17–19] as well as unconventional autophagic secretion [20]. Longitudinal genomic surveillance studies have identified artemisinin resistance associated malarial gene loci [21^●^,22^●^,23^●^] with multiple genes including several belonging to phosphoinositide pathways and autophagic pathways. A vesicular/autophagic resistancemechanism may be effective against new drugs that induce toxicity through protein aggregates or ‘proteopathy’ [24^●●^,^25^] and may present many targets such as plasmepsins also detected in the vesicle proteome [11^●●^], to facilitate resistance to partner drugs such as recently reported in multi drug resistant malaria [26,27]. However, a caveat to the ‘autophagy hypothesis’ is that PI3P vesicles in K13 mutants contain single membranes lacking luminal staining, while auto-phagosomes are double membraned and contain cellular components targeted for degradation. ER autophagy remains poorly understood in malaria parasites [28,29] and its mechanistic analyses need further study in artemisinin resistance.

## Artemisinin resistance mechanism of widespread parasite remodeling and host engagement

**Figure 4:**
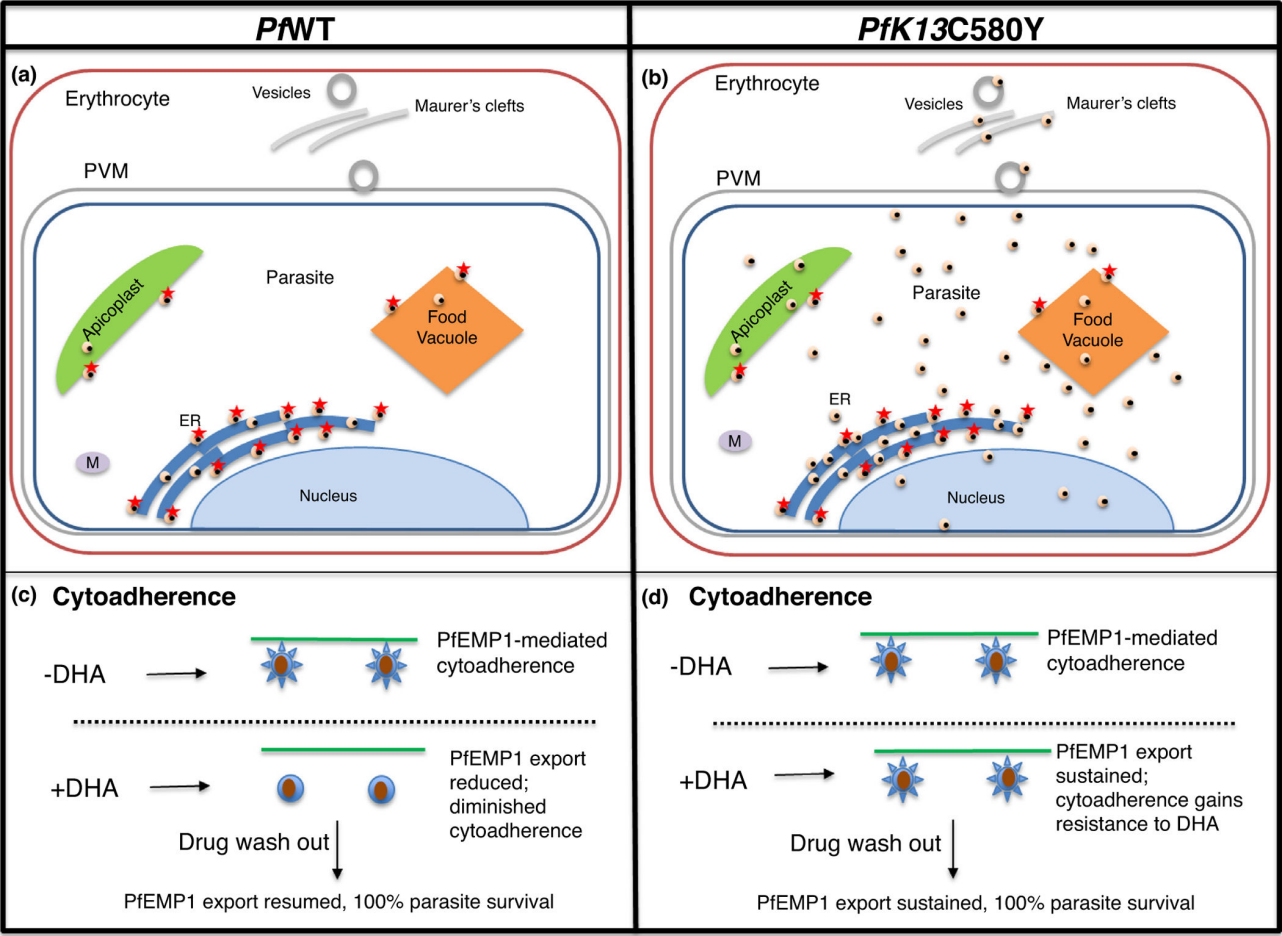
Expansion of PI3P vesicles by the major K13C580Y mutation of artemisinin resistance. (a) In *Plasmodium falciparum* wild type (WT) cells, PI3P vesicles (black in yellow spheres) with associated K13 (red star) are seen at parasite ER, apicoplast and food vacuole. (b) In the major mutation of artemisinin resistance K13C580Y, PI3P vesicles are amplified exported from the ER and disseminated in all organelles throughout the parasite as well as the erythrocyte, where PI3P is detected on vesicles and ‘Maurer’s clefts’ known to mediate the export of virulence determinants such as a major adhesin family (PfEMP1) to infected host cell surface. (c) Export of PfEMP1 (blue spikes) and cytoadherence of infected erythrocytes (blue circles with brown spheres) to host receptors (green line) are diminished by dihydroartemisinin (DHA) which is known to reduce levels of PI3P in artemisinin sensitive parasites [9,11^●●^]. (d) Export of PfEMP1 and cytoadherence are not diminished by DHA in artemisinin resistant parasites that show elevation in PI3P vesicles in the parasite and erythrocyte [11^●●^].

An unexpected feature of PI3P vesicles is that upon amplification they reach all destinations in mutant parasite (Figure 4a and b). This may explain complexity of artemisinin resistance as well as how a single K13-proteo- static determinant may reflect selective pressure in multiple organellar systems and hundreds of parasite genes implicated in resistance [2^●●^,3^●●^,^30^–^36^]. Since PI3P elevation is sufficient to induce resistance, expansion of PI3P vesicles may also account for K13-independent resistance [37^●^]. Another intriguing feature of PI3P vesicle expansion in resistant parasites is that the lipid also targets the red cell (Figure 4b) suggesting host factors may modulate artemisinin resistance [11^●●^]. Consistent with findings that PI3P is detected in intra-erythrocytic vesicles and Maurer’s clefts utilized in parasite protein export (Figure 4b), K13 mutations regulate the dynamics of export of PfEMP1 (the major parasite virulence determinant) to the host red cell. The K13 vesicle-proteome contains host-targeted protein translocation export (‘PTEX’) machinery [11^●●^,^38^,^39^] and several cargo with relevant export signals [40–42]. Whether all exported cargo utilize this pathway is not known: export to the host cell is complex and there may be steps of export selectivity that remain poorly understood [43–45]. Nonetheless dissemination of PI3P vesicles of proteostasis may also provide mechanisms to mitigate artemisinin damage to the host red cell. Finally, PfEMP1 export and cytoad- herence are inhibited by dihydroartemisinin in wild type parasites but both processes become resistant to drug in K13 mutants (Figure 4c, d, [11^●●^]). This suggests that in addition to protecting the infected red cell from proteopathy, K13 resistance mutations may provide better adherence to host receptors even in presence of drug. This may contribute to immune protection by helping the parasite avoid the spleen at both high and low parasite burdens. Additionally, K13 mutations may facilitate persistence of resistant parasites seen at low and asymptomatic parasitemias despite mass drug administration [46,47]. Since artemisinins and ACTs remain critical tools for malaria elimination, it will be important to understand how resistance enhances survival mechanisms within the parasite as well as the parasite’s engagement with its host.

## Conclusions

On the heels of the identification of K13 resistance marker, different technological approaches of transcriptomics (revealing associative UPR responses) versus functional and genetic studies (showing requirement of the lipid PI3P), suggested two distinct processes of artemisinin resistance. However, subsequent work using proteomics as an important bridging technology, suggests that both function in the same pathway. Furthermore, individual ER-UPR components are involved in artemisinin induced latency, but do not induce significant levels of resistance. Other candidate genes have not been tested. However, expansion of PI3P vesicles efficiently induces a log increase in artemisinin resistance. Vesicle formation and turnover are complex processes that may remodel both the parasite and red cell, suggesting that understanding their functions may provide insights about parasite mechanisms to survive exposure to artemisinins as well as host pathogenesis.
